# From anatomical knowledge to clinical practice: technical insights into dorsal metacarpal artery and perforator flaps – two case reports

**DOI:** 10.1097/RC9.0000000000000349

**Published:** 2026-03-09

**Authors:** Elise Lupon, Tanguy Perraudin, Pharel Njessi, Olivier Camuzard

**Affiliations:** a Department of Plastic, Reconstructive and Hand Surgery, University Institute of Locomotor and Sport (IULS), Pasteur Hospital, Nice, France; b Université Côte d’Azur, CNRS, LP2M, Nice, France

**Keywords:** anatomy, case report, dorsal metacarpal artery perforator flap, hand surgery, perforator flaps

## Abstract

**Introduction and importance:**

Advances in cutaneous vascular anatomy, including the perforasome concepts, have transformed hand reconstruction by enabling thin, pliable perforator flaps that preserve major axial vessels. These flaps may offer advantages over traditional fasciocutaneous designs. Safe and effective use depends on understanding perforator anatomy and adhering to key technical principles.

**Case presentation:**

Two cases illustrate the clinical implications of perforator-based reconstruction using the same flap design in its traditional non-perforator and perforator-based variants.

A 56-year-old man sustained a chainsaw laceration over the proximal interphalangeal joint of the fifth finger of the left hand. A traditional dorsal metacarpal artery flap was elevated in the subaponeurotic plane, requiring aponeurotic opening, division of intertendinous juncturae, and sacrifice of the fourth dorsal metacarpal artery. Coverage was achieved, but the flap was bulky, and donor-site healing was delayed.

A 43-year-old man with a dorsal laceration of the third finger of the left hand from broken glass was reconstructed using a dorsal metacarpal artery perforator flap elevated superficial to the dorsal interosseous aponeurosis. The perforator was found at its expected location, the pedicle was preserved with a perivascular adipose cuff, rotation was tension free, and donor-site closure was primary. Healing was uneventful.

**Clinical discussion:**

Perforator flaps may provide thinner, more adaptable tissue with reduced donor-site morbidity. Key principles include avoiding complete microskeletonization, preserving perivascular adipose tissue, and respecting gliding planes. Cadaveric training enhances anatomical understanding and reliability.

**Conclusion:**

These cases illustrate the potential role and technical interest of perforator-based local flaps for dorsal digital reconstruction in selected clinical situations.

## Introduction

For many years, hand reconstruction relied primarily on traditional fasciocutaneous flaps, whose design followed the classical principles of regional vascular territories. This approach was profoundly transformed by the anatomical work of Taylor and colleagues, who introduced the angiosome concept and mapped the body’s cutaneous vascular territories with unprecedented precision^[^1,2^]^. The subsequent development of the perforasome theory by Saint-Cyr further refined this understanding, demonstrating the dynamic interconnections, linking vessels, and perfusion patterns that support modern perforator flap design^[^3^]^. Together, these advances reshaped the foundations of cutaneous vascular anatomy and paved the way for perforator-based reconstruction.

HIGHLIGHTS

- Detailed anatomical knowledge of hand perforators is essential for selecting thin, reliable local flaps that preserve major vascular axes.
- Cadaveric training, including fixed and pulsatile reperfused models, enhances three-dimensional understanding of perforator pathways and surgical safety.
- A traditional dorsal metacarpal artery flap demonstrated greater donor-site morbidity and bulk compared with its perforator-based alternative.
- Comparative case analysis supports the growing use of perforator-based local flaps for dorsal digital reconstruction.

As a result, surgical practice progressively shifted toward thinner, more pliable, and better-vascularized flaps that preserve major axial vessels while reducing donor-site morbidity. These refinements have directly influenced hand surgery, where the need for delicate, contour-adapted coverage and preservation of tendon gliding surfaces makes perforator-based solutions particularly relevant. Perforator flaps may provide advantages over traditional hand flaps, potentially offering thinner and more pliable tissue with reliable vascularity and reduced donor site morbidity, which explains their growing adoption in reconstructive hand surgery^[^4^]^. Their mastery relies on several essential elements that include a solid understanding of perforator anatomy, meticulous dissection avoiding unnecessary skeletonization of the pedicle, and repeated cadaveric training to appreciate three-dimensional vascular pathways and tissue behavior^[^5^]^. Experience with larger caliber perforator flaps also strengthens technical confidence and precision when subsequently handling the smaller and more delicate perforators encountered in the hand^[^6^]^.

To illustrate the clinical relevance of these anatomical considerations, two cases using the same flap design in its traditional non-perforator form and its perforator-based version are presented in accordance with the updated SCARE guidelines^[^7^]^.

## Case presentation

**Figure 1.**
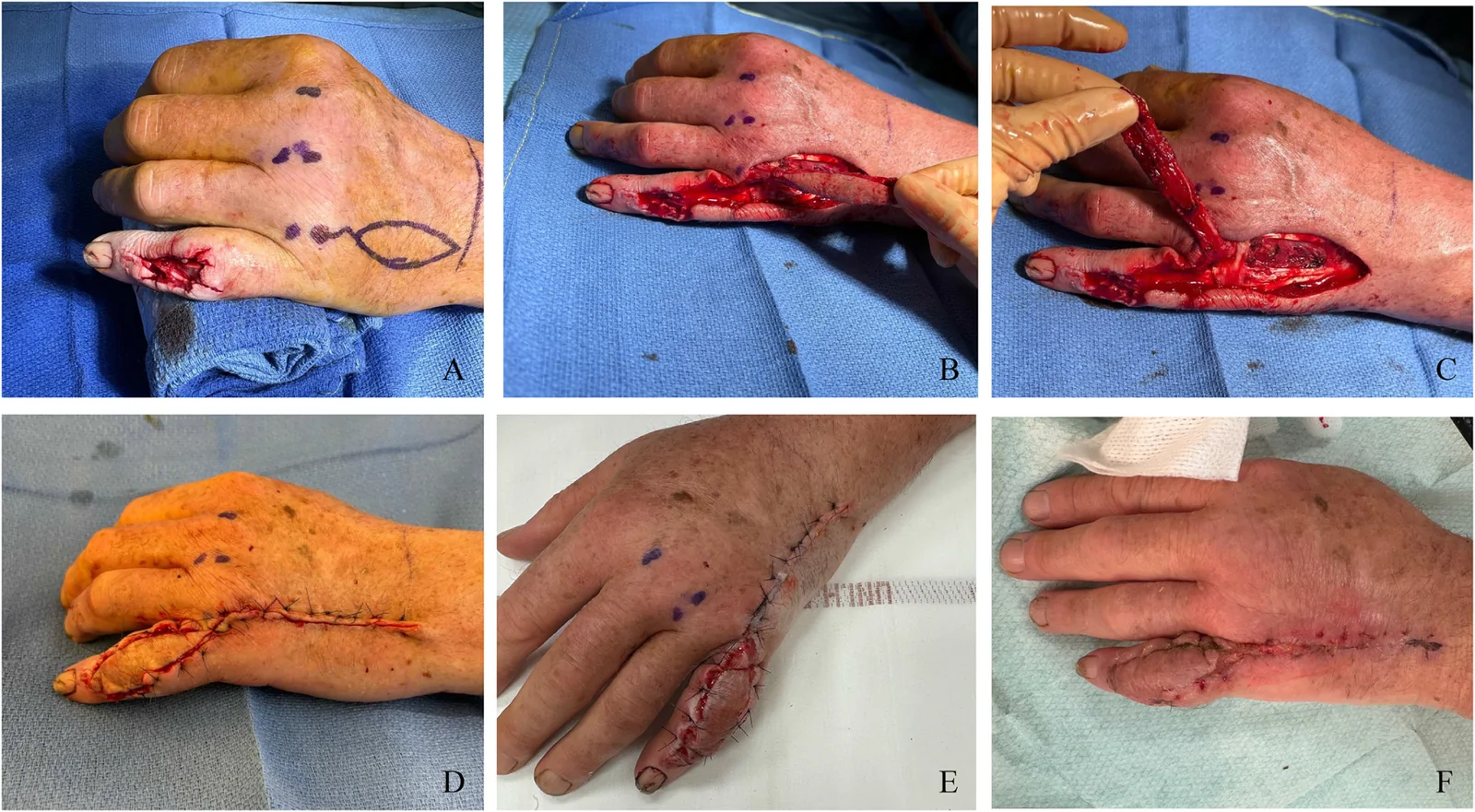
Classic dorsal metacarpal artery (DMCA) flap. (A) Dorsal defect over the fifth finger demonstrating exposure of the extensor tendon rupture at the level of the proximal interphalangeal (PIP) joint and along the middle phalanx. (B) Elliptical design of the fasciocutaneous flap based on the fourth DMCA, which at this level courses deep to the dorsal aponeurosis. (C) Elevation of the DMCA fasciocutaneous flap showing donor-site morbidity, including opening of the dorsal aponeurosis, dissection between the fourth and fifth extensor tendons to harvest the artery, division of intertendinous juncturae, and removal of the interosseous aponeurosis containing the vascular pedicle. (D) Immediate postoperative view after flap mobilization. (E) Postoperative appearance on day 2 after DMCA flap inset, demonstrating the relatively thick nature of the fasciocutaneous flap. (F) Postoperative appearance on day 15 after suture removal, showing delayed donor-site healing consistent with mild vascular compromise due to sacrifice of the fourth intermetacarpal artery. *Printed with permission; copyrights retained by Elise Lupon, MD, PhD.*

The first case involved a right-hand dominant 56-year-old man who presented to the emergency hand unit with a dorsal laceration of the left hand caused by a chainsaw at the level of the proximal interphalangeal joint and extending across the middle phalanx. He smoked approximately 20 cigarettes per day and was under treatment for depression, with no other relevant medical history. Radiographs showed no abnormalities. Surgical exploration revealed a laceration of the superficial terminal slip of the extensor tendon. After debridement of devitalized skin and copious irrigation with normal saline, a dorsal exposure of the repaired extensor mechanism was evident. A traditional dorsal metacarpal artery (DMCA) flap was elevated based on the fourth DMCA, which at this level courses deep to the dorsal aponeurosis, to provide coverage of the proximal interphalangeal joint and the middle phalanx of the fifth finger of the left hand. The DMCA flap was raised in the subaponeurotic plane, necessitating opening of the dorsal aponeurosis, dissection between the fourth and fifth extensor tendons to isolate the artery, division of the intertendinous juncturae, and removal of the interosseous aponeurosis containing the vascular pedicle. The flap was secured with loose simple interrupted non-absorbable sutures, and the patient was discharged home after the procedure. The total operative time, including extensor tendon repair and flap elevation, was 60 minutes. He received 48 hours of antibiotic prophylaxis because of the traumatic open injury with joint exposure, in accordance with institutional protocols for open hand trauma. He was treated with an extension splint for 4 weeks. Stitches were removed at 2 weeks. Postoperative findings demonstrated adequate defect coverage, although the flap exhibited a relatively bulky dorsal contour over the fifth finger and delayed donor-site healing consistent with the morbidity induced by sacrificing the fourth DMCA (Fig. 1). Postoperatively, delayed donor-site healing was observed, without flap necrosis or infection. The complication was managed conservatively with local wound care and healed completely within 4 weeks, without functional sequelae. Clinical follow-up at 9 months demonstrated complete wound healing, stable soft-tissue coverage, preserved finger motion, and a satisfactory functional outcome.

**Figure 2.**
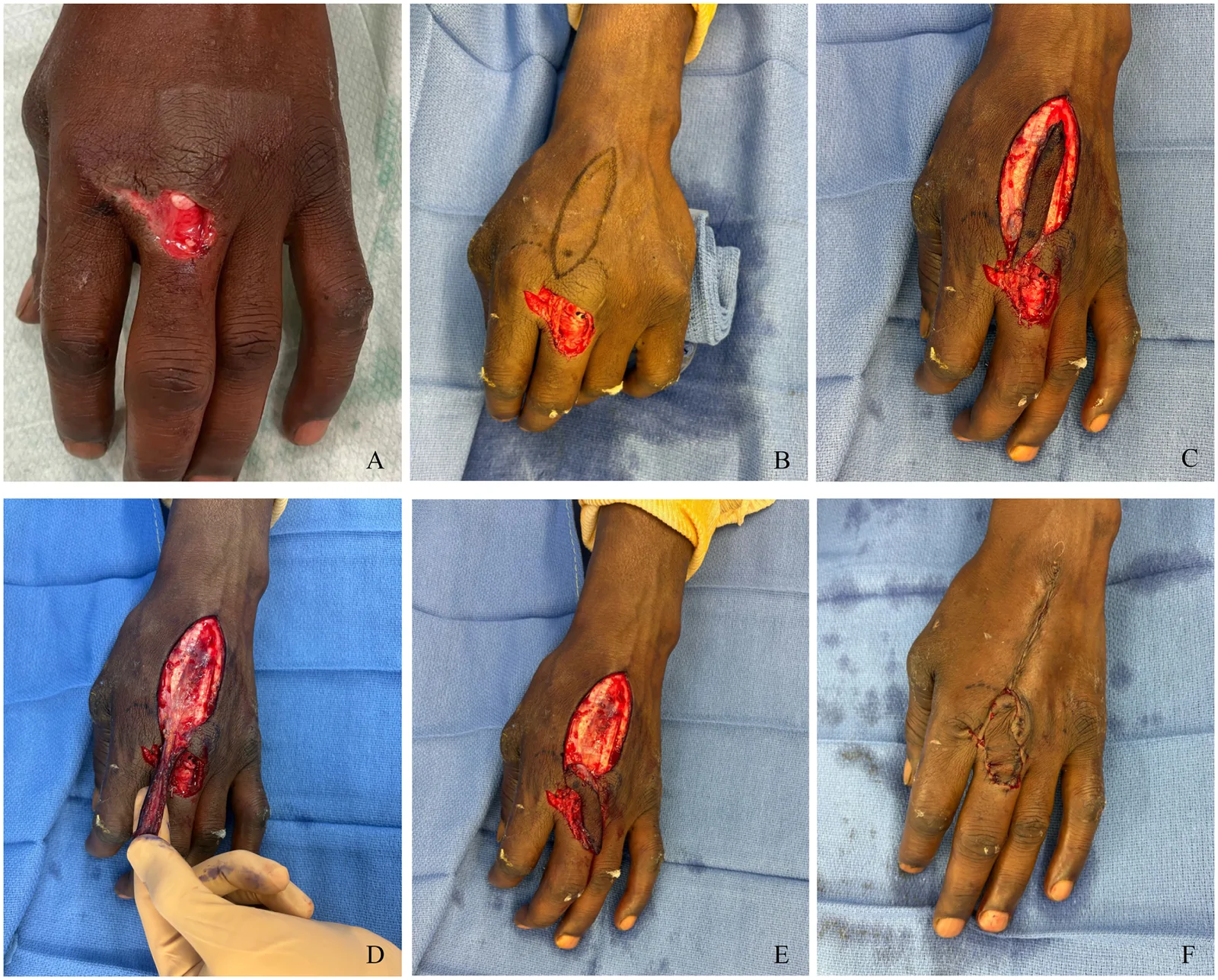
Dorsal metacarpal artery perforator (DMAP) flap for extensor tendon coverage. (A) Dorsal defect over the third finger demonstrating the complete extensor tendon rupture at the level of the metacarpophalangeal joint. (B) Extensor tendon repair using 4-0 non-absorbable U-sutures. Elliptical design of the perforator flap based on the second intermetacarpal perforator. The vascular anatomy at this level is highly constant, making preoperative Doppler mapping unnecessary. (C) Superficial skin incision along the flap design, showing continuity of the dorsal aponeurosis. (D) Intraoperative view of the flap elevated superficial to the dorsal interosseous aponeurosis and the extensor peritendinous plane, with identification of the DMCA perforator pedicle. Complete microskeletonization of the pedicle, separating the artery and vein, is unsafe in the hand; maintaining a cuff of perivascular adipose tissue is recommended to preserve pedicle integrity and reduce the risk of vasospasm or injury. (E) Intraoperative view showing preservation of the dorsal interosseous aponeurosis and peritendinous plane. Flap rotation of approximately 160° was achieved without fascial release or inclusion of excessive peripedicular fat. (F) Postoperative appearance on day 2 after inset of the DMCA perforator flap, demonstrating tension-free closure and a satisfactory dorsal contour. *Printed with permission; copyrights retained by Elise Lupon, MD, PhD.*

By contrast, the perforator-based variant generally allows thinner and more pliable tissue with reduced donor site morbidity. In the second case, a DMCA perforator flap was used to cover a complete extensor tendon repair at the dorsal aspect of the third finger. A right-hand dominant 43-year-old man with no medical history other than active smoking (1 pack-year) sustained a laceration over the dorsal aspect of the intermetacarpal region of his left hand caused by broken glass. Anteroposterior and lateral radiographs showed no fracture. No preoperative Doppler or ultrasound mapping was performed. The joint was thoroughly irrigated with saline using a catheter needle, followed by extensor tendon repair using 4-0 non-absorbable U-sutures. A superficial skin incision was made along the flap design, preserving continuity of the dorsal aponeurosis, and the flap was then elevated superficial to the dorsal interosseous aponeurosis and the extensor peritendinous plane, with identification of the DMCA perforator pedicle. The dissection of the pedicle maintained a cuff of perivascular adipose tissue. The perforator was found at its expected location in the second intermetacarpal space, consistent with the high reproducibility described in anatomical series^[^4^]^. Elevation was performed above the dorsal interosseous fascia in order to preserve both this layer and the peritendinous plane, thereby maintaining tendon gliding surfaces. A protective cuff of perivascular adipose tissue was kept around the perforator pedicle throughout the elevation to avoid complete microskeletonization. The flap rotated into the defect without tension, and the donor site was closed primarily with resorbable suture. The total operative time, including extensor tendon repair and perforator flap elevation, was 45 minutes. The patient was discharged home after the procedure and healing proceeded without complications. He was treated with an extension splint for 4 weeks. A 48-hour course of antibiotic prophylaxis was administered due to the traumatic nature of the injury and articular exposure, following accepted institutional practice for open hand injuries. Stitches were removed at 2 weeks, and no donor-site complications were observed. The long-term result demonstrated a supple dorsal contour, a thinner and better adapted reconstruction, no donor-site issues, and complete functional recovery (Fig. 2). At 7 months of follow-up, healing was complete, with full range of motion of the involved finger and no functional limitation.

## Discussion

The introduction of perforator flaps has significantly expanded reconstructive possibilities, allowing surgeons to achieve thinner, more pliable, and better-adapted tissue coverage while preserving major axial vessels and reducing donor-site morbidity^[^4,8–10^]^. Their use is particularly advantageous in the hand, where reconstruction must respect delicate gliding planes and restore contour with minimal bulk. As anatomical knowledge has improved through detailed cadaveric studies and clinical series, perforator-based flaps have become increasingly reliable options in dorsal digital reconstruction^[^4,11^]^.

However, several technical principles remain essential to ensure their safety and reproducibility. Complete microskeletonization of the pedicle, separating the artery and vein, is unsafe in the hand because of the small caliber and fragility of digital perforators. Maintaining a cuff of perivascular adipose tissue around the pedicle is recommended to preserve vascular integrity and minimize the risk of vasospasm or iatrogenic injury during rotation or inset^[^5^]^. This protective approach is consistent with observations made in both clinical and anatomical models, where pedicle handling directly influences perfusion stability^[^12–14^]^.

Respecting the dorsal interosseous aponeurosis and the extensor peritendinous plane is also crucial for optimal outcomes, as these structures contribute to maintaining tendon gliding and digital motion. When these principles are correctly applied, perforator-based local flaps may provide thin, contour-adapted tissue with fewer donor-site sequelae than traditional DMCA flaps, a benefit highlighted in both our cases and previous literature^[^4^]^.

A detailed understanding of hand perforator anatomy is fundamental for selecting reliable local flaps that preserve the major vascular axes of the hand while providing thin and well-adapted soft tissue coverage. Recent anatomical studies have refined our knowledge of both dorsal and palmar perforators, including the constant branches of the dorsal metacarpal system and the more variable perforators arising from common digital and superficial palmar branches^[^4^]^. These data contribute to improving local reconstructive options and may reduce the need for more extensive or morbid flap transfers.

Most anatomical mapping has historically been carried out on formalin fixed cadavers, a methodology that facilitates layer by layer exposure but alters tissue elasticity and causes vessel collapse, potentially underestimating perforator caliber and behavior under physiological conditions^[^15^]^. We favor the use of fresh cadaveric material stored at 4°C for training, as this preserves tissue characteristics closer to the clinical situation^[^16–19^]^. Fresh pulsatile reperfused cadaver models also provide complementary information, allowing dynamic assessment of perforator filling, flap pliability, and pedicle responsiveness during manipulation or rotation^[^20^]^. The combination of fixed tissue dissection and training on reperfused models offers the most complete anatomical and functional perspective for understanding hand perforator systems.

**Figure 3.**
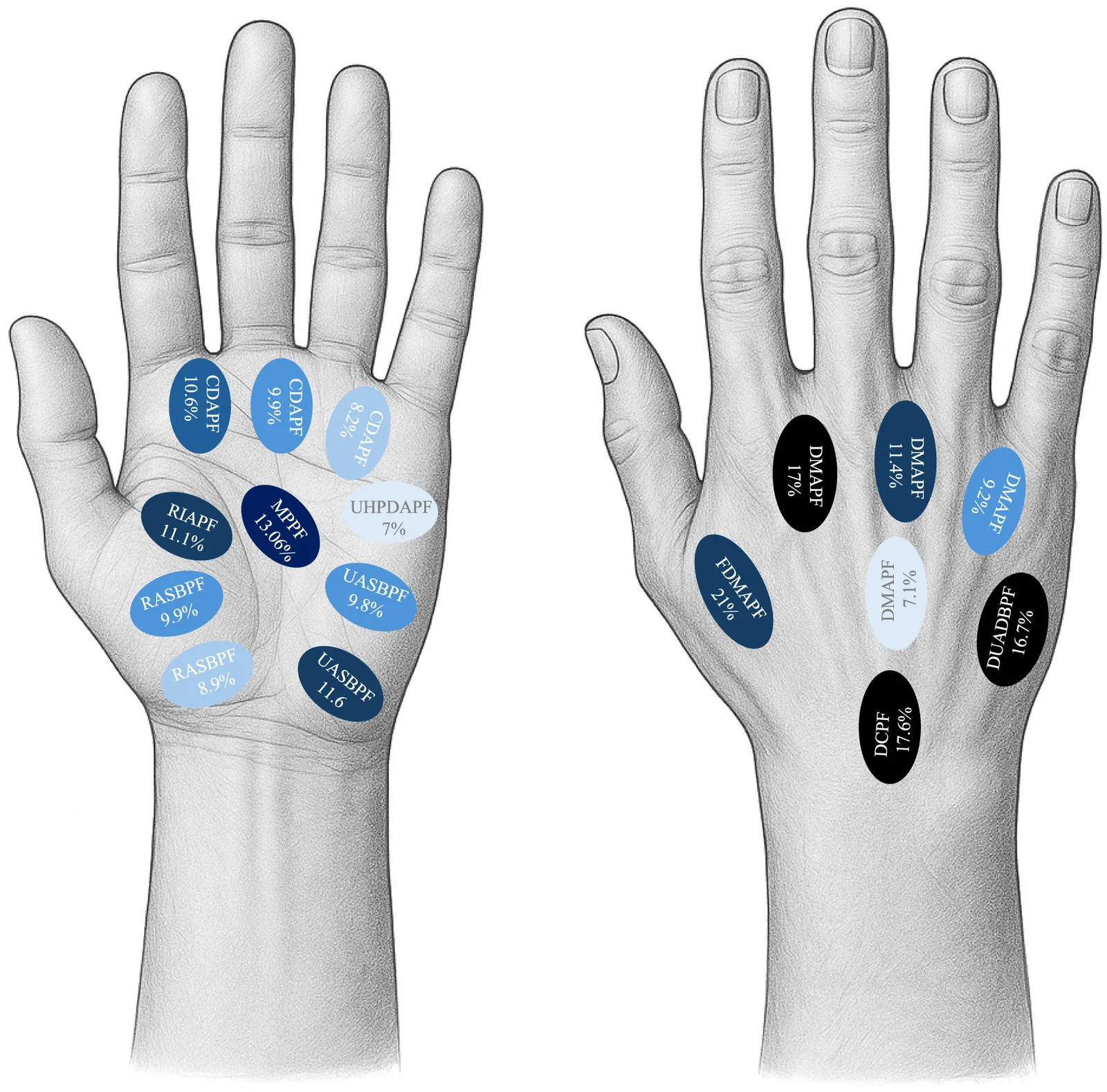
Summary illustrations annotated with clinically relevant dorsal and palmar perforators, illustrating their density and distribution across hand subregions. The relative distribution percentages are derived from previously published anatomical studies^[^4^]^. The darker blue regions indicate areas with a higher density of perforators, whereas the lighter blue regions correspond to areas with lower perforator density. Left: Names and proportions of palmar perforators relative to the total palmar surface. Palmar perforator flaps include RASPBP flap (radial artery superficial palmar branch perforator), UASPBP flap (ulnar artery superficial palmar branch perforator), CDAP flap (common digital artery perforator), RIAP flap (radialis indicis artery perforator), MPP flap (mid-palmar perforator), and UHPDAP flap (ulnar hypothenar palmar digital artery perforator). Right: Names and proportions of dorsal perforators relative to the total dorsal surface. Dorsal perforator flaps include DMAP flap (dorsal metacarpal artery perforator), FDMAP flap (first dorsal metacarpal artery perforator), DUADBP flap (dorsal ulnar artery distal branch perforator), and DCP flap (dorsal carpal perforator). *Printed with permission; copyrights retained by Elise Lupon, MD, PhD.*

Although dorsal perforators are better documented, palmar vascular territories remain less represented in the literature despite their reconstructive potential. Regions such as the proximal hypothenar, distal thenar, and mid palmar compartments contain reproducible perforators that may allow defect adjacent palmar flaps while preserving the main axial vessels^[^21^]^. A schematic representation of the main dorsal and palmar perforator distributions is provided, using the standardized nomenclature defined by the Tokyo Consensus Conference on Perforator Flaps^[^22^]^ and the distributions described in anatomical studies^[^4^]^, to facilitate preoperative planning when thin local tissue is required (Fig. 3).

Operative time is often cited as a potential drawback of perforator-based flaps, particularly during the learning curve in the hand where perforators are small. In our experience, however, a DMCA perforator flap does not meaningfully increase operative time compared with a classic DMCA flap. The traditional DMCA technique requires subaponeurotic dissection with aponeurotic opening, deeper pedicle isolation, and management/closure of these planes, which can be time consuming. Once anatomical landmarks are mastered and perforator dissection principles are applied (limited pedicle handling, preservation of a perivascular adipose cuff), DMAP elevation can be performed efficiently while preserving the major vascular axis and minimizing donor-site morbidity.

Flap selection in these cases was not influenced by smoking status. Although both patients were active smokers, no predefined threshold of cigarette consumption was used to guide the choice between a traditional DMCA flap and its perforator-based variant. To date, there is no evidence-based cutoff of tobacco exposure that contraindicates the use of hand perforator flaps. In the second case, no preoperative Doppler or ultrasound mapping was performed, as DMCA perforators are known to be highly constant and can be reliably identified intraoperatively in experienced hands. The use of a traditional DMCA flap in the first case and a perforator-based variant in the second reflects an evolution in surgical strategy and increasing familiarity with hand perforator anatomy rather than patient-related selection criteria.

Integrating anatomical data derived from both cadaver models and clinical experience reinforces the value of perforator-based local flaps in hand reconstruction. These options provide reliable and thin coverage with minimal donor site morbidity while preserving the functional and aesthetic integrity of the hand, particularly in dorsal digital defects where maintenance of tendon gliding planes is essential.

A limitation of this report is the absence of late postoperative photographic documentation for the second case at a time point comparable to the first case. While clinical follow-up was performed, standardized photographs were not available. This limits direct visual comparison at later stages of healing. These cases are presented for illustrative and educational purposes, and no direct comparative conclusions should be inferred.

## Conclusions

Perforator-based dorsal metacarpal flaps may provide thin, reliable soft-tissue coverage with fewer donor-site complications than traditional designs. Knowledge of perforator anatomy and adherence to key technical principles are essential for safe and effective use in hand reconstruction.

## Data Availability

The data supporting the findings of this study are included within the article. No additional datasets were generated or analyzed during the current study.

## Research registration unique identifying number (UIN)

Name of the registry: IORG0012275 - iULS-University Institute for Locomotion and Sport. Unique identifying number or registration ID: IRB00014528_2025_48.

## SCARE guideline

The work has been reported in line with the SCARE 2025 criteria.
